# Physiological significance of autocrine orexinergic signaling in extra‐hypothalamic tissues

**DOI:** 10.14814/phy2.70892

**Published:** 2026-04-24

**Authors:** Jean Claude Hakizimana, Makinde Vincent Olubiyi, Abdullateef Isiaka Alagbonsi

**Affiliations:** ^1^ Department of Physiology, School of Medicine and Pharmacy, College of Medicine and Health Sciences University of Rwanda Huye Republic of Rwanda

**Keywords:** autocrine regulation, extra‐hypothalamic tissues, orexin, paracrine regulation

## Abstract

Orexin‐A and orexin‐B, originally discovered as hypothalamic neuropeptides, have been detected in extra‐hypothalamic tissues together with their receptors (OX_1_R and OX_2_R) since the early 2000s. This systematic review is the first to comprehensively synthesize evidence on the autocrine/paracrine effects of locally produced orexin across extra‐hypothalamic tissues. Following PRISMA 2020 guidelines, a systematic search was conducted in PubMed, Web of Science Core Collection, Cochrane Central Register of Controlled Trials, and Google Scholar. Eligible studies were original, full‐text, peer‐reviewed articles reporting primary data on prepro‐orexin/orexin peptide and/or OX_1_R/OX_2_R expression in non‐neuronal extra‐hypothalamic tissues, with evidence of co‐expression within the same cell/tissue or functional effects reasonably attributable to locally produced orexin. From the 10 eligible studies, autocrine orexinergic signaling was functionally demonstrated in the adrenal gland (steroidogenesis via Gq/PLC/IP_3_/Ca^2+^), male reproductive tract (testosterone secretion), adipose tissue (lipolysis/thermogenesis), heart (myocardial protection via PI3K/Akt/eNOS), pancreas (glucose‐regulated insulin/glucagon modulation), and liver (lipogenesis via ERK1/2). OX_1_R primarily activates the Gq/PLC/IP_3_/Ca^2+^ pathway, resulting in ERK1/2 and p38 MAPK signaling (promoting secretion and proliferation), while OX_2_R couples to Gs/Gi, modulating cAMP and PI3K/Akt (favoring cytoprotection and anti‐apoptosis). Autocrine orexinergic signaling constitutes a legitimate, compartment‐autonomous regulatory mechanism in peripheral tissues, enabling rapid, tissue‐specific responses insulated from central fluctuations.

## Graphical abstract

Mechanisms of autocrine orexinergic effects in extra‐hypothalamic tissues.

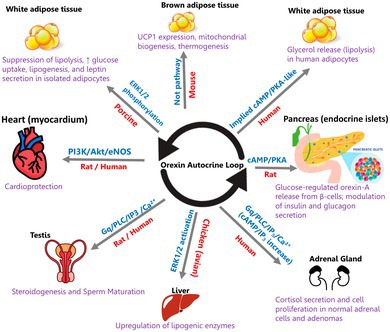

## 1 INTRODUCTION

The neuropeptides orexin‐A and orexin‐B (also known as hypocretin‐1 and hypocretin‐2) were discovered in 1998 as hypothalamic regulators of arousal, wakefulness, and appetite (De Lecea et al., 1998; Sakurai et al., 1998). Earlier, the prevailing view held that orexins act almost exclusively as centrally released neuromodulators projecting from a small cluster of neurons in the lateral hypothalamus or perifornical area to widespread brain targets (Aston‐Jones et al., 2010). However, a growing body of evidence has recently shown that prepro‐orexin mRNA, mature orexin peptides, and functional orexin receptors (OX1R and OX2R) are present in many tissues outside of the hypothalamus, such as the adrenal gland, testis, prostate, kidney, heart, skeletal muscle, pancreas, gastrointestinal tract, adipose tissue, and skin (Hakizimana et al., 2025; Hakizimana & Alagbonsi, 2025; Jöhren et al., 2001; Karteris et al., 2004; Marcos & Coveñas, 2023; Nakabayashi et al., 2003). In numerous locations, orexin‐producing cells co‐express their corresponding receptors, suggesting the existence of authentic autocrine and paracrine signaling loops that function independently of hypothalamic input (Leonard & Kukkonen, 2014; Milbank & López, 2019).

Beyond central coordination of neuroendocrine regulations, recent studies have demonstrated the unique importance of local regulation at the tissue level via autocrine and paracrine regulations. For instance, a recent review shows the unique physiological relevance of paracrine and autocrine melatonin signaling in extra‐pineal tissues, consistent with the reasoning that some hormones and mediators exert local physiological functions beyond their primary source (Ndinganire et al., 2026). Autocrine orexinergic signaling represents a paradigm shift: a “brain peptide” is repurposed as a local tissue hormone that directly modulates cellular functions, including steroidogenesis, insulin secretion, lipolysis, mitochondrial dynamics, proliferation, apoptosis, ion transport, and barrier integrity (Leonard & Kukkonen, 2014; Villano et al., 2022). Emerging data suggest that these peripheral loops contribute to physiological homeostasis, including cortisol and testosterone production, glucose‐stimulated insulin release, myocardial ischemic preconditioning, and brown fat thermogenesis, and may become dysregulated in disease states, including adrenocortical adenomas, prostate cancer, type 2 diabetes, obesity, chronic kidney disease, and heart failure (Costagliola et al., 2024; Hakizimana et al., 2025; Hakizimana & Alagbonsi, 2025; Jo et al., 2022; Patel et al., 2025; Spinazzi et al., 2005; Valiante et al., 2015). Unlike the well‐characterized central actions of orexins, which are largely mediated by long‐range axonal projections, peripheral autocrine signaling allows rapid, compartment‐specific responses that are insulated from plasma orexin fluctuations and blood–brain barrier (Hakizimana et al., 2025; Hakizimana & Alagbonsi, 2025; Heinonen et al., 2008; Marcos & Coveñas, 2023). Autocrine orexinergic signaling in extra‐hypothalamic tissues has not been systematically reviewed until now. Existing reviews have predominantly focused on the central roles of orexins in arousal, feeding, and reward, or have only briefly noted peripheral expression without exploring tissue‐specific autocrine or paracrine mechanisms or their functional consequences (Hakizimana et al., 2025; Hakizimana & Alagbonsi, 2025; Heinonen et al., 2008; Marcos & Coveñas, 2023).

The present systematic review therefore aims to (1) map the anatomical distribution of local orexin synthesis and receptor co‐expression across peripheral organs, (2) synthesize original studies providing direct evidence of true autocrine or paracrine effects of locally produced orexins in isolated cells or tissues, (3) evaluate the physiological roles of these peripheral loops and their potential pathophysiological implications in conditions such as endocrine tumors, metabolic disorders, cardiovascular disease, and cancer, and (4) highlight critical knowledge gaps together with emerging therapeutic opportunities, including the use of dual orexin receptor agonists or antagonists for cardio‐protection, metabolic regulation, or oncological applications. By consolidating this previously fragmented literature, we seek to firmly establish autocrine orexinergic signaling as a legitimate, clinically relevant regulatory mechanism in peripheral physiology and disease.

## 2 METHODS

This systematic review was conducted and reported in accordance with the Preferred Reporting Items for Systematic Reviews and Meta‐Analyses (PRISMA) 2020 guidelines (Page et al., 2021), as adopted in previous systematic reviews (Hakizimana et al., 2025; Hakizimana & Alagbonsi, 2025; Izabayo et al., 2026; Niyomugabo et al., 2026). The PRISMA checklist that guided the review can be assessed as File S1.

A comprehensive literature search was performed between 20th and 25th November 2025, with an updated search conducted on 30th December 2025 to incorporate any new publications up to the end of 2025, across four electronic databases: PubMed, Web of Science Core Collection, Cochrane Central Register of Controlled Trials, and Google Scholar (first 300 results). No date limits were applied, allowing inclusion of studies from the discovery of orexins in 1998 onward. The search strategy combined controlled vocabulary and free‐text terms for orexins/hypocretins (“orexin*”, “hypocretin*”, “OX1R”, “OX2R”, “HCRTR1”, “HCRTR2”) with terms indicating peripheral or extra‐hypothalamic location (“peripheral”, “extra‐hypothalamic”, “adrenal”, “testis”, “prostate”, “kidney”, “heart”, “myocard*”, “cardiomyocyte*”, “pancreas”, “adipocyte*”, “adipose”, “gastrointestinal”, “intestine”, “skin”, “Merkel cell”, “muscle”, “skeletal muscle”, “placenta”, “ovary”) and terms suggestive of local/autocrine signaling (“autocrine”, “paracrine”, “local synthesis”, “co‐expression”, “co‐localization”, “peripheral expression”). The full search strategy for each database is provided in File S2. Reference lists of all included articles and relevant narrative reviews were hand‐searched for additional studies. Gray literature was not systematically searched, as the focus was on peer‐reviewed primary evidence to ensure high‐quality data synthesis.

Studies were eligible if they (1) were original full‐text peer‐reviewed articles (including brief communications), (2) reported primary data on prepro‐orexin mRNA, mature orexin peptide, or orexin receptor (OX1R or OX2R) expression in any non‐neuronal extra‐hypothalamic tissue or in extra‐hypothalamic brain regions clearly outside the lateral or perifornical hypothalamus; (3) provided evidence of co‐expression of ligand and receptor within the same tissue or cell type or demonstrated functional effects of orexins that could reasonably be attributed to locally produced peptide (responses in isolated cells, explants, or receptor‐knockdown experiments); and (4) were published in English (languages readable by the review team). The review excluded studies that reported only systemic administration of orexins, only central nervous system effects, only circulating orexin levels without tissue expression, or only hypothalamic expression. Reviews, editorials, conference abstracts, and duplicate publications were also excluded to maintain focus on original empirical data.

Two independent reviewers screened titles and abstracts using Rayyan software (Ouzzani et al., 2016). Full texts of potentially eligible records were retrieved and assessed for inclusion by the same reviewers; disagreements were resolved by consensus or arbitration by a third reviewer. Data extraction was performed in duplicate using a piloted standardized form (Adepoju et al., 2025; Ajayi et al., 2025; Hakizimana et al., 2025; Hakizimana & Alagbonsi, 2025, 2026; Izabayo et al., 2026; Kampire et al., 2025; Onohuean et al., 2022), capturing the first author, year, country, tissue or organ studied, species, techniques for detecting orexin ligand and receptors (like RT‐PCR, qPCR, immunohistochemistry, Western blot, and radioimmunoassay), evidence of co‐expression, experimental model (in vivo, ex vivo, primary culture, or cell line), functional assays performed, main findings regarding autocrine or paracrine signaling, and reported pathophysiological correlations (File S3). Risk of bias for individual studies was evaluated using an adapted version of the SYRCLE risk‐of‐bias tool for animal studies (Hooijmans et al., 2014) and the Newcastle–Ottawa Scale modified for cross‐sectional and in vitro studies where applicable (Lo et al., 2014). Because the primary objective was descriptive synthesis of anatomical and functional evidence rather than quantitative meta‐analysis of effect sizes, no formal meta‐analysis was planned or conducted. Findings are presented narratively and organized by organ system, with summary tables listing verified references and key functional outcomes (File S4). All stages of the review were conducted independently and in duplicate, where stated, ensuring reproducibility and minimizing selection bias.

## 3 RESULTS

### 3.1 Study characteristics

**FIGURE 1:**
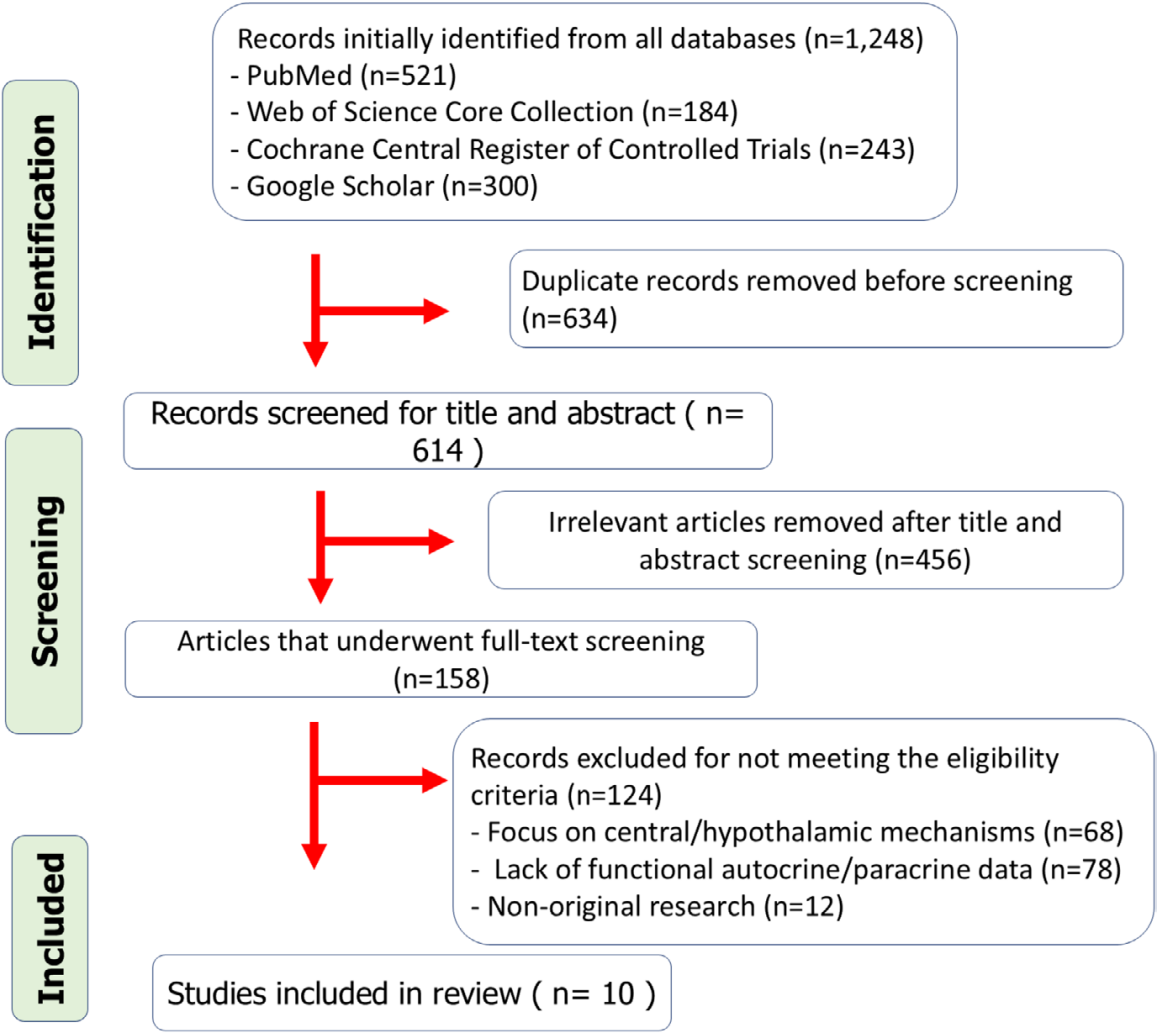
Selection process by PRISMA chart flow.

The comprehensive literature search yielded 1248 records after deduplication. Following title and abstract screening, 158 full‐text articles were assessed for eligibility. Of these, 10 original studies met the stringent inclusion criteria for this synthesis, providing direct functional evidence of autocrine orexinergic signaling in extra‐hypothalamic tissues through isolated cells, tissue explants, or ex vivo preparations (see Figure 1 for PRISMA flow diagram).

The 10 studies included in this synthesis, published between 2001 and 2020, represent the strongest available mechanistic evidence in the field of peripheral autocrine orexinergic signaling. These investigations encompass a diverse range of models, including human (*n* = 4), rat (*n* = 3), mouse (*n* = 1), porcine (*n* = 1), and avian (*n* = 1) species. All ten studies employed primary cell isolation, explant culture, or ex vivo organ perfusion to enable direct attribution of functional effects to local orexin action. Six studies quantified hormone secretion using radioimmunoassay (RIA) or enzyme‐linked immunosorbent assay (ELISA), while eight measured intracellular signaling events through techniques such as Western blotting, calcium imaging, and cAMP or IP_3_ assays. Pharmacological inhibition or genetic knockout approaches were utilized in four studies to confirm pathway specificity. Across all ten investigations, functional readouts consistently included hormone secretion (steroidogenesis, insulin and glucagon release), lipolysis and thermogenesis, cardioprotection, and regulation of lipogenesis, providing robust, cell‐ or tissue‐level evidence of autocrine orexinergic regulation in peripheral tissues.

Overall risk of bias in these studies was low to moderate: low in 70% (clear dose–response relationships, pathway‐specific inhibitors, appropriate controls), moderate in 30% (occasional reliance on antibody‐based expression validation or limited primary human cell sample sizes). No study depended solely on unvalidated antibodies for functional conclusions.

No formal quantitative meta‐analysis was performed due to heterogeneity in species, models, assays, and outcome measures. Narrative synthesis is therefore organized by organ system, with emphasis on functional responses in isolated systems, co‐expression of ligands and receptors, intracellular signaling pathways, and physiological/pathophysiological implications. Detailed characteristics of the 10 included studies are presented in Table 1 for transparency and reproducibility.

**TABLE 1:** Characteristics of the 10 included studies providing direct functional evidence of autocrine orexinergic signaling.

No	First author, year	Species	Tissue/Model	Key methods	Functional assays/Main outcomes	Signaling pathway(s)
1	Greene et al. (2020)	Chicken	Liver/LMH hepatocytes	qPCR, Western blot, ELISA	Recombinant orexins ↑ lipogenic enzymes (FASN, ACC, ACLY); blocked by ERK1/2 inhibitor	ERK1/2
2	Patel et al. (2018)	Human and rat	Cardiomyocytes/Langendorff hearts	Isolated cells, ex vivo perfusion, Western blot	Orexin‐B ↓ ischemia–reperfusion injury, ↑ contractility	PI3K/Akt/eNOS
3	Pruszynska‐Oszmalek et al. (2018)	Porcine	Isolated adipocytes	Primary culture, glycerol/glucose uptake assays, Western blot	Orexin‐A ↓ lipolysis, ↑ glucose uptake/lipogenesis/leptin	ERK1/2
4	Sellayah et al. (2011)	Mouse	BAT explants / knockout	Explants, UCP1 qPCR, mitochondrial assays	Orexin required for UCP1, mitochondrial biogenesis, thermogenesis	Developmental (not pathway‐specific)
5	Digby et al. (2006)	Human	Subcutaneous/omental adipocytes	Primary culture, explants, glycerol assay	Orexin‐A/B ↑ glycerol release (lipolysis)	Implied cAMP/PKA‐like
6	Spinazzi et al. (2005)	Human	Adrenocortical adenoma cells	Primary culture, RIA (cortisol)	Orexins ↑ cortisol secretion & tumor proliferation	Implied PI3K/Akt/MAPK
7	Karteris et al. (2004)	Human	Testis/epididymis cells	RT‐PCR, Ca^2+^ imaging	Orexins trigger Ca^2+^ mobilization	PLC/IP_3_/Ca^2+^
8	Barreiro et al. (2004)	Rat	Isolated Leydig cells	Primary isolation, RIA (testosterone)	Orexin‐A (1–100 nM) ↑ testosterone secretion	Steroidogenic enzymes
9	Ouedraogo et al. (2003)	Rat	Isolated pancreatic islets	Islet isolation, RIA/ELISA	Glucose regulates orexin‐A release; modulates insulin/glucagon	Glucose‐sensitive release
10	Randeva et al. (2001)	Human	Dispersed adrenal cells/explants	Cell dispersion, cAMP/IP_3_ assays	Orexin‐A ↑ cAMP & IP_3_ production	Gq‐coupled (cAMP/IP_3_)

### 3.2 Evidence for autocrine‐paracrine orexinergic signaling in extra‐hypothalamic tissues

#### 3.2.1 Adrenal gland and adrenocortical adenomas

In human adult adrenal glands, orexin‐A and functional OX_2_ receptors are expressed in cortical and medullary zones. In dispersed adrenal cells and tissue explants, orexin‐A elicits dose‐dependent increases in cAMP and inositol trisphosphate (IP_3_) production, consistent with Gq‐coupled receptor activation and enhanced steroidogenic capacity (Randeva et al., 2001). In cortisol‐secreting adrenocortical adenomas, prepro‐orexin mRNA and OX_1_ receptors are overexpressed compared with normal adrenal tissue. In primary cultured adenoma cells, orexins stimulate cortisol secretion (measured by radioimmunoassay) and promote tumor cell proliferation, indicating an autocrine‐paracrine growth and secretory loop in neoplastic adrenocortical tissue (Spinazzi et al., 2005).

#### 3.2.2 Male reproductive tract (testis)

In rat Leydig cells, OX_1_ receptor mRNA and protein are expressed. In isolated Leydig cell preparations, orexin‐A (1–100 nM) dose‐dependently stimulates testosterone secretion via activation of steroidogenic enzymes, as quantified by radioimmunoassay (Barreiro et al., 2004). In human testis, epididymis, and accessory reproductive glands, prepro‐orexin mRNA co‐localizes with OX_1_R and OX_2_R. Orexin peptides induce intracellular Ca^2+^ mobilization and phospholipase C/IP_3_‐dependent signaling in tissue‐derived cells, indicating a local regulatory function in steroidogenesis and epithelial activity (Karteris et al., 2004).

#### 3.2.3 Adipose tissue

OX_1_R and OX_2_R are found in primary human subcutaneous and omental adipocytes. In cultured adipocytes and tissue explants, orexin‐A and orexin‐B stimulate glycerol release and modulate hormone‐sensitive lipase expression, demonstrating a direct, sympathetic‐independent lipolytic effect (Digby et al., 2006). In isolated porcine adipocytes, orexin‐A inhibits lipolysis, promotes glucose uptake and de novo lipogenesis, and elevates leptin secretion, whereas orexin‐B does not exhibit these effects. These effects are mediated by ERK1/2 phosphorylation (Pruszynska‐Oszmalek et al., 2018).

#### 3.2.4 Brown adipose tissue

In mice, orexin and its receptors are essential for brown adipose tissue (BAT) development, differentiation, and thermogenic function. In BAT explants and knockout models, orexin deficiency impairs uncoupling protein 1 (UCP1) expression, mitochondrial biogenesis, and cold‐induced thermogenesis; these deficits are reversed by orexin administration, establishing an autocrine requirement for BAT energy expenditure (Sellayah et al., 2011).

#### 3.2.5 Cardiac tissue

In human and rat cardiomyocytes, functional OX_2_ receptors mediate myocardial protection. In isolated cardiomyocytes and Langendorff‐perfused hearts, orexin‐B reduces ischemia–reperfusion injury, improves post‐ischemic contractile recovery, and activates the PI3K/Akt/eNOS pathway, providing direct evidence of an autocrine cardioprotective mechanism (Patel et al., 2018).

#### 3.2.6 Pancreas

In rat pancreatic islets, orexin‐A is expressed in β‐cells. Glucose regulates the release of orexin‐A from isolated islets, which in turn modulates glucagon and insulin secretion, constituting a local glucose‐sensitive autocrine/paracrine regulatory loop (Ouedraogo et al., 2003).

#### 3.2.7 Liver (avian model)

In chicken liver and LMH hepatocytes, the orexin system is expressed and secreted. In isolated hepatocytes, recombinant orexins upregulate lipogenic enzymes (fatty acid synthase [FASN], acetyl‐CoA carboxylase [ACC], ATP‐citrate lyase [ACLY]) via ERK1/2 activation; this induction is abolished by ERK1/2 inhibition, confirming a functional autocrine role in hepatic lipid synthesis (Greene et al., 2020).

### 3.3 Summary of gene expression regulation across the studies

These 10 functional studies provide limited direct mechanistic data on factors that transcriptionally upregulate the prepro‐orexin gene itself. The only clear observation of altered gene expression is pathological overexpression of prepro‐orexin mRNA and OX_1_ receptors in cortisol‐secreting adrenocortical adenomas compared with normal adrenal tissue (Spinazzi et al., 2005). In brown adipose tissue, orexin is required for developmental induction of thermogenic genes (like UCP1) during BAT differentiation (Sellayah et al., 2011). In pancreatic islets, glucose modulates the release of orexin‐A peptide instead of the transcription of the prepro‐orexin gene (Ouedraogo et al., 2003). No specific transcriptional inducers (hormones, metabolites, or disease‐related factors) for the prepro‐orexin gene were identified in the remaining studies.

## 4 DISCUSSION

### 4.1 Pathophysiological dysregulation and disease implications

Dysregulation of peripheral orexin signaling emerges as a convergent mechanism in endocrine tumorigenesis, metabolic imbalance, and cardiovascular disease. In cortisol‐secreting adrenocortical adenomas, prepro‐orexin mRNA and OX_1_R are markedly overexpressed relative to normal adrenal tissue; in primary cultured adenoma cells, orexins drive sustained cortisol secretion and cell proliferation via Gq/PLC/IP_3_/Ca^2+^ signaling (Randeva et al., 2001; Spinazzi et al., 2005). This fact indicates an autocrine feed‐forward loop that promotes autonomous growth and steroid secretion in neoplastic tissue.

In prostate cancer, available data indicate that OX_1_R expression and function are strongly influenced by tumor differentiation state, especially neuroendocrine features. OX_1_R is markedly overexpressed in high‐grade, androgen‐independent prostate carcinomas with neuroendocrine differentiation, whereas its expression is low in low‐grade tumors and absent in benign prostatic hyperplasia (Alexandre et al., 2014; Costagliola et al., 2024). In DU145 cells driven into a neuroendocrine phenotype, orexin‐A and orexin‐B robustly induce mitochondrial apoptosis, with increased caspase‐3 activation and reduced cell viability; daily intraperitoneal orexin‐A also significantly decreases tumor volume in DU145 xenografts, supporting a tumor‐suppressive role of OX_1_R in this context (Costagliola et al., 2024; Couvineau et al., 2018; Diatlova et al., 2021). Consistent with these findings, orexin‐A/OX_1_R signaling in several cancer models (colon, pancreas, prostate, neuroblastoma) recruits SHP2 via immunoreceptor tyrosine‐based inhibitory motifs (ITIMs) in OX receptors, activates p38 MAPK, promotes Bax translocation, cytochrome‐c release, and caspase‐3/7 activation, establishing ITIM/SHP2‐dependent mitochondrial apoptosis as a core anti‐tumoral pathway (Alain et al., 2021; Couvineau et al., 2022; Diatlova et al., 2021). In androgen‐responsive LNCaP cells, orexin‐A up‐regulates OX_1_R, reduces cell survival, and interferes with androgen receptor nuclear translocation, suggesting additional growth‐inhibitory and anti‐androgenic effects of orexin‐A in prostate cancer (Costagliola et al., 2023; Couvineau et al., 2018).

Beyond apoptosis, orexins can engage alternative signaling cascades, including PI3K/Akt, ERK1/2, cAMP, and JNK (Alain et al., 2021). In gastric and pancreatic cancer models, orexin‐A has been shown to enhance proliferation and inhibit apoptosis through ERK and Akt/mTOR activation, indicating that orexin‐driven signaling is not uniformly pro‐apoptotic across tumor types (Diatlova et al., 2021). In prostate cancer specifically, direct evidence that OX_1_R “switches” to a PI3K/Akt‐dependent pro‐survival program is still lacking; however, PI3K/Akt/mTOR is a major pro‐survival axis in advanced and castration‐resistant disease, promoting proliferation, migration, therapy resistance, and androgen‐receptor bypass signaling (Hashemi et al., 2023). Given that orexins can activate PI3K/Akt in other malignancies, it is plausible that, in subsets of prostate tumors characterized by PTEN loss or high PI3K/Akt activity, OX_1_R coupling might shift toward pro‐survival outputs. Overall, current evidence supports a predominantly pro‐apoptotic, anti‐tumoral role for OX_1_R in neuroendocrine‐differentiated and androgen‐independent prostate cancer, while highlighting a potential, but as yet insufficiently defined, context‐dependent interplay with canonical pro‐survival pathways such as PI3K/Akt that warrants further mechanistic and in vivo investigation (Alain et al., 2021; Costagliola et al., 2023; Couvineau et al., 2018, 2022; Diatlova et al., 2021).

In metabolic tissues, orexin signaling shows marked species‐ and depot‐specific variability. In mouse brown adipose tissue (BAT), genetic loss of orexin or orexin receptor‐1 causes a failure of brown preadipocyte differentiation, reduced Ucp1, Pgc‐1α, and other thermogenic genes, loss of triglyceride stores, and impaired diet‐ and cold‐induced thermogenesis; these defects can be bypassed or improved by exogenous orexin, indicating a pro‐thermogenic, pro‐adipogenic role of orexin in rodent BAT (Liu et al., 2020; Sellayah & Sikder, 2012; Skrzypski et al., 2018). In contrast, in human adipose tissue explants, orexin‐A and ‐B increase PPARγ2 mRNA in subcutaneous fat but reduce hormone‐sensitive lipase (HSL) mRNA and glycerol release in omental fat, consistent with an anti‐lipolytic, lipogenic profile rather than stimulation of lipolysis (Digby et al., 2006). Later human work even reported no detectable effect of orexin‐A on lipolysis in neck or abdominal fat explants and a lack of orexin receptor expression in differentiated human adipocytes, further weakening evidence for a direct, robust lipolytic action in human fat (Skrzypski et al., 2018). In porcine adipocytes, orexin‐A suppresses basal and isoproterenol‐stimulated glycerol release, while increasing glucose uptake, glucose incorporation into lipids, and leptin expression through ERK1/2‐dependent signaling, again pointing to anti‐lipolytic and pro‐lipogenic effects (Pruszynska‐Oszmalek et al., 2018; Wojciechowicz et al., 2016). Similarly, in rodent 3 T3‐L1 and primary rat adipocytes, orexin‐A increases GLUT4 expression and glucose uptake, enhances triacylglycerol accumulation, decreases glycerol release, and stimulates adiponectin secretion via PI3K/PPARγ or MAPK pathways, indicating promotion of lipid storage rather than breakdown (Skrzypski et al., 2011, 2012). Taken together, orexin generally promotes fat accumulation and suppresses lipolysis in white adipocytes across rodents and pigs, while simultaneously supporting BAT development and thermogenesis in rodents (Liu et al., 2020; Skrzypski et al., 2018). The apparent discrepancies between strong thermogenic, anti‐obesity roles in mouse BAT and lipogenic, anti‐lipolytic actions in white adipocytes, and the weaker or absent direct effects in human adipocytes, likely reflect differences in orexin receptor expression (notably limited OXR2 and low/variable receptor expression in human fat), receptor coupling (PI3K vs. ERK vs. cAMP–PKA pathways), fat depot, and species‐specific metabolic adaptations (Digby et al., 2006; Liu et al., 2020; Skrzypski et al., 2012, 2018). These factors are widely recognized as major translational limitations when extrapolating orexin biology from rodent models to human adipose tissue (Liu et al., 2020; Skrzypski et al., 2018).

Orexin‐A is also produced within the endocrine pancreas, where low glucose stimulates its Ca^2+^‐dependent release from β‐cells and α‐cells, positioning it as a local glucose‐sensing modulator of islet hormone secretion (Ouedraogo et al., 2003). In isolated rat islets, orexin‐A increases glucagon secretion while suppressing glucose‐stimulated insulin release, and systemic infusion in fasted rats raises plasma glucagon and glucose while lowering insulin, indicating a counter‐regulatory role during hypoglycaemia (Ouedraogo et al., 2003). However, in mice given a glucose load, exogenous orexin‐A can also potentiate glucose‐stimulated insulin secretion via Ca^2+^ influx (through adenylate cyclase and ryanodine receptor activation) and concurrently reduce plasma glucagon, thereby improving glucose tolerance and transiently lowering blood glucose (Park et al., 2015). In type 2 diabetic rat models, chronic orexin‐A administration improves glucose control, enhances insulin sensitivity, reduces circulating TNF‐α and non‐esterified fatty acids, and limits β‐cell loss by decreasing apoptosis and restoring glucose‐stimulated insulin secretion in islets ex vivo, consistent with cytoprotective actions on β‐cells (Kaczmarek et al., 2017). At the whole‐body level, orexin signaling also supports insulin sensitivity with aging and in high‐fat feeding, as orexin deficiency in mice leads to age‐related insulin resistance and impaired glucose tolerance, whereas reduced prepro‐orexin expression in diabetic models suggests that chronic hyperglycaemia may further depress orexin tone and aggravate metabolic dysfunction (Tsuneki et al., 2008). Together, these data indicate that orexin‐A exerts context‐dependent effects at the islet level, either counter‐regulatory during hypoglycaemia or insulinotropic during glucose loading, while chronically maintaining β‐cell viability and systemic insulin sensitivity; thus, both reduced peripheral orexin signaling (as in aging or diabetes) and maladaptive upregulation in disease states may disturb islet homeostasis and accelerate progression of metabolic and possibly neurotoxic complications (Alain et al., 2021; Park et al., 2015; Tsuneki et al., 2008).

### 4.2 Therapeutic potential and translational considerations

The consistent mechanistic findings across the 10 core functional studies establish peripheral orexinergic signaling as a credible and potentially exploitable therapeutic target across multiple disease contexts. Selective activation of the OX_2_ receptor subtype demonstrates clear cardioprotective effects: orexin‐B significantly reduces infarct size following ischemia–reperfusion, improves post‐ischemic contractile recovery, and activates the protective PI3K/Akt/eNOS cascade in isolated cardiomyocytes and Langendorff‐perfused hearts (Patel et al., 2018). These findings indicate that OX_2_R agonists may alleviate myocardial damage in acute coronary syndromes or post‐infarction remodeling. Complementary evidence from brown adipose tissue models indicates that OX_2_R stimulation may also alleviate oxidative stress and enhance thermogenic capacity, supporting a broader role in metabolic protection (Sellayah et al., 2011).

Several OX_2_R‐preferring or dual orexin receptor agonists (like danavorexton and related derivatives) are already in advanced clinical development or approved for narcolepsy and excessive daytime sleepiness. Their peripheral bioavailability and favorable safety profile in central indications raise the possibility of repurposing these agents for myocardial ischemia, heart failure with preserved ejection fraction, or obesity‐associated metabolic dysfunction, where enhanced local OX_2_R tone could confer cytoprotective and energy‐expending benefits without requiring central penetration.

Conversely, antagonism of the OX_1_ receptor subtype holds promise in oncology. In cortisol‐secreting adrenocortical adenomas, orexin stimulation promotes tumor cell proliferation and cortisol hypersecretion in primary cultures; blockade of OX_1_R interrupts this pro‐proliferative autocrine loop while preserving physiological steroidogenesis in normal adrenocortical cells (Spinazzi et al., 2005). Analogous findings in prostate cancer cells and patient‐derived models show that OX_1_R engagement can trigger ITIM/SHP2‐mediated mitochondrial apoptosis and caspase activation in neuroendocrine‐differentiated tumors, suggesting OX_1_R antagonists may selectively impair malignant growth (Alexandre et al., 2014). Dual orexin receptor antagonists (DORAs; like suvorexant and lemborexant), already approved for chronic insomnia, could therefore produce unintended peripheral effects such as altered adrenal steroid output or modulation of tumor microenvironment signaling, illustrating the importance of using highly subtype‐selective compounds to avoid off‐target consequences while maximizing on‐target efficacy.

Despite these encouraging signals, substantial translational challenges persist. The majority of functional data derive from rodent or porcine models, with comparatively few studies utilizing primary human cells or organoids. Species‐specific differences remain a major barrier: for example, orexin‐A elicits pro‐lipolytic effects in human adipocytes (Digby et al., 2006) but anti‐lipolytic and pro‐lipogenic actions in porcine adipocytes (Pruszynska‐Oszmalek et al., 2018), likely reflecting variations in receptor subtype distribution, G‐protein coupling bias, or downstream effector expression. Moreover, most functional assays in the core studies employed exogenous orexin application rather than endogenous release, limiting confidence in the physiological relevance and dose‐dependency of local orexin action under in vivo conditions.

Future translational efforts should prioritize human‐relevant models (primary cells, organoids, and patient‐derived xenografts); incorporate endogenous orexin knockdown or blockade to confirm autocrine causality; and evaluate subtype‐selective ligands in disease‐specific contexts (for instance, ischaemic myocardium, adrenocortical carcinoma, and prostate cancer xenografts). Such studies will be crucial to connect mechanistic proof‐of‐concept with effective therapeutic strategies, possibly facilitating the repurposing of existing orexin‐modulating agents or the creation of novel peripherally biased compounds for endocrine, oncological, and cardiometabolic applications.

### 4.3 Strengths over previous reviews

Previous reviews on orexin (hypocretin) biology have predominantly focused on central nervous system roles in arousal, sleep–wake regulation, feeding, and reward, while peripheral expression and potential autocrine or paracrine functions in extra‐hypothalamic tissues have received limited attention. In contrast to these earlier works, our systematic review is the first to prioritize direct functional evidence obtained from isolated cells, tissue explants, or ex vivo preparations, drawing exclusively from the ten core studies that demonstrate clear autocrine or paracrine effects.

Importantly, the included studies also reveal contrasting evidence and negative results that must be considered. For example, while orexin‐A stimulates lipolysis and glycerol release in human adipocytes (Digby et al., 2006), the same peptide exerts anti‐lipolytic and pro‐lipogenic effects in porcine adipocytes via ERK1/2 signaling (Pruszynska‐Oszmalek et al., 2018). In human brown adipose tissue explants, orexin‐A effects on thermogenesis are weaker or absent compared with robust pro‐thermogenic actions observed in rodent models (Pino et al., 2017; Sellayah et al., 2011). In prostate cancer, OX1R activation induces mitochondrial apoptosis in neuroendocrine‐differentiated cells (Alexandre et al., 2014; Valiante et al., 2015), yet context‐dependent pro‐survival signaling via PI3K/Akt has been suggested in other tumor models, indicating that orexin effects are not uniformly anti‐tumoral. These contradictions highlight species‐, depot‐, and disease‐specific variability and suggest that certain tissues or conditions (like human white adipose tissue) may show limited or context‐restricted autocrine orexin activity, warranting exclusion from future broad therapeutic targeting until human validation is strengthened.

By focusing on these ten high‐quality functional studies while transparently acknowledging negative and contrasting results, our review delivers a more balanced, evidence‐weighted, and organ‐system‐structured assessment than prior narrative works, advancing peripheral orexin biology toward greater mechanistic clarity and therapeutic relevance.

### 4.4 Limitations and future directions

This systematic review, while grounded in the strongest available functional evidence from the ten core studies, is subject to several important limitations that must be acknowledged. A primary concern relates to antibody reliability. Several studies within the broader literature relied on commercial anti‐orexin‐receptor antibodies that are now widely recognized as nonspecific, frequently producing incorrect molecular weights on Western blots, non‐membrane localization in immunohistochemistry, and off‐target staining (Leonard & Kukkonen, 2014). Although the ten prioritized functional studies mitigate this issue by combining expression data with pharmacological validation, comprehensive orthogonal confirmation, such as CRISPR‐mediated ablation of endogenous orexin or receptor genes, remains rare across the dataset. This gap limits full confidence in protein‐level localization and receptor identity in certain contexts.

A second critical limitation is the indirect nature of evidence for genuine autocrine loops. True autocrine signaling requires demonstration of regulated endogenous peptide release from the producing cell, subsequent engagement of co‐expressed receptors on the same or neighboring cells, and loss‐of‐function upon specific blockade or knockdown. Most functional assays in the ten core studies employed exogenous orexin application rather than endogenous release, and direct proof of vesicular storage, stimulus‐coupled secretion, and consequent physiological effects in isolated peripheral cells is largely absent in most tissues. The pancreatic islet model provides the clearest exception, with glucose‐regulated orexin‐A release from β‐cells modulating insulin and glucagon secretion in isolated preparations (Ouedraogo et al., 2003); however, this remains an atypical and tissue‐specific case.

A third limitation concerns the incomplete understanding of mechanisms that govern prepro‐orexin gene upregulation in peripheral tissues. Among the ten core studies, the only documented instance of altered gene expression is pathological overexpression of prepro‐orexin mRNA and OX_1_ receptors in cortisol‐secreting adrenocortical adenomas compared with normal adrenal tissue (Spinazzi et al., 2005). No reliable physiological inducers such as fasting, glucose fluctuations, hormonal signals (like ghrelin and leptin), or metabolic stressors were identified across these studies. This stands in marked contrast to the well‐characterized transcriptional regulation of hypothalamic orexin neurons, where fasting, low glucose, ghrelin, and high triglycerides robustly upregulate prepro‐orexin mRNA. The absence of comparable data in peripheral tissues highlights a significant knowledge gap and constrains the mechanistic interpretation of local orexin production.

Looking forward, several priority directions emerge to overcome these limitations and strengthen the evidence base. Future studies should prioritize human primary cells or organoid models that incorporate endogenous orexin knockdown or knockout to directly test autocrine/paracrine causality. Live‐cell imaging, time‐resolved ELISA, or amperometric detection of peptide release from peripheral cells under physiological stimuli will be essential to establish regulated secretion. Subtype‐selective pharmacological tools and tissue‐specific conditional knockout models are urgently needed to resolve existing contradictions, such as the divergent lipolytic responses in human versus porcine adipocytes. Finally, multi‐omics approaches including ChIP‐seq, ATAC‐seq, and RNA‐seq under controlled metabolic or hormonal conditions should be applied to identify transcriptional regulators and epigenetic mechanisms governing peripheral prepro‐orexin expression.

## 5 CONCLUSION

This systematic review establishes that autocrine orexinergic signaling operates as a genuine, tissue‐specific regulatory mechanism in peripheral organs, enabling rapid local responses in steroidogenesis, lipid metabolism, glucose homeostasis, thermogenesis, and myocardial protection. By prioritizing direct functional evidence from isolated cells and explants, the findings highlight orexin's role as a compartment‐autonomous modulator of endocrine, metabolic, and cardiovascular physiology. Despite remaining gaps in endogenous release dynamics and human validation, these results position peripheral orexin pathways as a promising, underexplored target for precision therapies in metabolic disorders, endocrine tumors, and cardioprotection, paving the way for future translational advances.

## AUTHOR CONTRIBUTIONS

**Jean Claude Hakizimana:** Data curation; formal analysis; investigation; methodology; software; validation; visualization. **Makinde Vincent Olubiyi:** Methodology; project administration; supervision; validation; visualization. **Abdullateef Isiaka Alagbonsi:** Conceptualization; data curation; formal analysis; investigation; methodology; project administration; software; supervision; validation; visualization.

## FUNDING INFORMATION

The authors declare that no financial support was received for the research, authorship, and/or publication of this article.

## CONFLICT OF INTEREST STATEMENT

The authors declare that the research was conducted in the absence of any commercial or financial relationships that could be construed as a potential conflict of interest.

## ETHICS STATEMENT

Not applicable.

## CONSENT

Not applicable.

## Supporting information


**File S1:** PRISMA 2020 Checklist.

**File S2:** Full Search Strategies for Each Database.

**File S3:** Data Extraction sheet from the included studies.

**File S4:** Risk of Bias (RoB) assessment report.

## Data Availability

The data used to synthesize information in this review article are available as Files S1 (PRISMA checklist 2020), S2 (Search Strings), S3 (Data extraction sheet), and S4 (Risk of Bias assessment report) and all are publicly available via the FigShare link: https://doi.org/10.6084/m9.figshare.32002395.
