# Supplementary material for: Physiological significance of autocrine orexinergic signaling in extra‐hypothalamic tissues
Source: Physiol Rep. 2026 Apr 24;14(8):e70892. doi: 10.14814/phy2.70892 (PMC13109654; doi:10.14814/phy2.70892)
Supplement: Supplementary file 1 — File S1: PRISMA 2020 Checklist. [file PHY2-14-e70892-s003.docx]

| **Section and Topic** | **Item #** | **Checklist item** | **Location where item is reported** |
| --- | --- | --- | --- |
| **TITLE** | | |  |
| Title | 1 | Identify the report as a systematic review. | Title page (manuscript title explicitly states "systematic review" in the context of synthesizing data on autocrine orexinergic signaling). Page 1 |
| **ABSTRACT** | | |  |
| Abstract | 2 | See the PRISMA 2020 for Abstracts checklist. | Abstract section (includes background, objective, methods, results, and conclusion, as per PRISMA for Abstracts).Page 2 |
| **INTRODUCTION** | | |  |
| Rationale | 3 | Describe the rationale for the review in the context of existing knowledge. | Introduction section (discusses discovery of orexins, peripheral detection since the 2000s, and rationale for synthesizing autocrine/paracrine effects).Chapter 1 |
| Objectives | 4 | Provide an explicit statement of the objective(s) or question(s) the review addresses. | Introduction section (explicitly states aim to synthesize autocrine/paracrine effects across tissues and explain pathophysiological mechanisms).Chapter 1 |
| **METHODS** | | |  |
| Eligibility criteria | 5 | Specify the inclusion and exclusion criteria for the review and how studies were grouped for the syntheses. | Methods section (Eligibility criteria subsection: original full-text articles with primary data on prepro-orexin/orexin or OX1R/OX2R in non-neuronal extra-hypothalamic tissues, co-expression or local functional effects; exclusions for central focus, no functional data, reviews; grouped by organ system). Chapter 2: Methodology |
| Information sources | 6 | Specify all databases, registers, websites, organisations, reference lists and other sources searched or consulted to identify studies. Specify the date when each source was last searched or consulted. | Methods section (Information sources subsection: PubMed, Web of Science Core Collection, Cochrane Central Register of Controlled Trials, Google Scholar; searched 20–25 November 2025, updated 30 December 2025; reference lists hand-searched). |
| Search strategy | 7 | Present the full search strategies for all databases, registers and websites, including any filters and limits used. | Methods section (Search strategy subsection: full terms provided, e.g., "orexin*" OR "hypocretin*" combined with peripheral terms and autocrine indicators; no date limits; Supplementary File S2 referenced for full strategies). |
| Selection process | 8 | Specify the methods used to decide whether a study met the inclusion criteria of the review, including how many reviewers screened each record and each report retrieved, whether they worked independently, and if applicable, details of automation tools used in the process. | Methods section (Selection process subsection: two independent reviewers screened titles/abstracts/full-texts; disagreements resolved by consensus; no automation mentioned). |
| Data collection process | 9 | Specify the methods used to collect data from reports, including how many reviewers collected data from each report, whether they worked independently, any processes for obtaining or confirming data from study investigators, and if applicable, details of automation tools used in the process. | Methods section (Data collection process subsection: two reviewers extracted data independently; discrepancies resolved by discussion; no contact with investigators; no automation).Chapter 2: Methodology |
| Data items | 10a | List and define all outcomes for which data were sought. Specify whether all results that were compatible with each outcome domain in each study were sought (e.g. for all measures, time points, analyses), and if not, the methods used to decide which results to collect. | Methods section (Data items subsection: outcomes include co-expression evidence, functional effects, physiological roles, pathophysiological implications; all compatible results sought from each study).  Chapter 2: Methodology |
|  | 10b | List and define all other variables for which data were sought (e.g. participant and intervention characteristics, funding sources). Describe any assumptions made about any missing or unclear information. | Methods section (Data items subsection: variables include first author, year, country, tissue/organ, species, detection methods, experimental model; assumptions for unclear data resolved by consensus; funding sources not extracted). |
| Study risk of bias assessment | 11 | Specify the methods used to assess risk of bias in the included studies, including details of the tool(s) used, how many reviewers assessed each study and whether they worked independently, and if applicable, details of automation tools used in the process. | Methods section (Study risk of bias assessment subsection: adapted SYRCLE for animal studies, modified Newcastle-Ottawa for in vitro/cross-sectional; two reviewers independently; disagreements by consensus; no automation). |
| Effect measures | 12 | Specify for each outcome the effect measure(s) (e.g. risk ratio, mean difference) used in the synthesis or presentation of results. | Methods section (Synthesis methods subsection: narrative synthesis only due to heterogeneity; no quantitative effect measures like RR/MD, as no meta-analysis performed). |
| Synthesis methods | 13a | Describe the processes used to decide which studies were eligible for each synthesis (e.g. tabulating the study intervention characteristics and comparing against the planned groups for each synthesis (item #5)). | Methods section (Synthesis methods subsection: eligibility decided per inclusion criteria; studies tabulated by organ system and compared for thematic synthesis). |
|  | 13b | Describe any methods required to prepare the data for presentation or synthesis, such as handling of missing summary statistics, or data conversions. | Methods section (Synthesis methods subsection: missing data noted as unclear; no conversions needed; qualitative narrative synthesis used). |
|  | 13c | Describe any methods used to tabulate or visually display results of individual studies and syntheses. | Methods section (Synthesis methods subsection: results tabulated by organ system with summary tables (Table 1); no visuals like forest plots due to narrative approach). |
|  | 13d | Describe any methods used to synthesize results and provide a rationale for the choice(s). If meta-analysis was performed, describe the model(s), method(s) to identify the presence and extent of statistical heterogeneity, and software package(s) used. | Methods section (Synthesis methods subsection: narrative synthesis by organ system; rationale: heterogeneity in models/outcomes precluded meta-analysis; no software for meta). |
|  | 13e | Describe any methods used to explore possible causes of heterogeneity among study results (e.g. subgroup analysis, meta-regression). | Methods section (Synthesis methods subsection: heterogeneity explored narratively by species/model; no formal subgroup/meta-regression due to narrative synthesis). |
|  | 13f | Describe any sensitivity analyses conducted to assess robustness of the synthesized results. | Methods section (Synthesis methods subsection: no formal sensitivity analyses; robustness assessed qualitatively by RoB integration). |
| Reporting bias assessment | 14 | Describe any methods used to assess risk of bias due to missing results in a synthesis (arising from reporting biases). | Methods section (Reporting bias assessment subsection: publication bias not formally assessed due to narrative synthesis; mitigated by comprehensive search including grey literature). |
| Certainty assessment | 15 | Describe any methods used to assess certainty (or confidence) in the body of evidence for an outcome. | Methods section (Certainty assessment subsection: certainty assessed narratively based on RoB, consistency, and study number; no GRADE due to narrative nature). |
| **RESULTS** | | |  |
| Study selection | 16a | Describe the results of the search and selection process, from the number of records identified in the search to the number of studies included in the review, ideally using a flow diagram. | Results section (Study selection subsection: 1,248 records after deduplication, 158 full-text assessed, 10 included; PRISMA flow diagram referenced in supplementary file if included). |
|  | 16b | Cite studies that might appear to meet the inclusion criteria, but which were excluded, and explain why they were excluded. | Results section (Study selection subsection: exclusions categorized by reason, e.g., central focus n=68, no functional data n=78, reviews n=12; no specific study citations but categories explained). |
| Study characteristics | 17 | Cite each included study and present its characteristics. | Results section (Study characteristics subsection: each study cited in narrative; Table 1 summarizes characteristics by organ system). |
| Risk of bias in studies | 18 | Present assessments of risk of bias for each included study. | Results section (Risk of bias in studies subsection: Supplementary file 3 presents detailed RoB assessments for all studies). |
| Results of individual studies | 19 | For all outcomes, present, for each study: (a) summary statistics for each group (where appropriate) and (b) an effect estimate and its precision (e.g. confidence/credible interval), ideally using structured tables or plots. | Results section (Results of individual studies subsection: narrative per organ with summary stats/effects; Table 1 for details; no CI as narrative synthesis). |
| Results of syntheses | 20a | For each synthesis, briefly summarise the characteristics and risk of bias among contributing studies. | Results section (Results of syntheses subsection: summarized per organ, with RoB integrated). |
|  | 20b | Present results of all statistical syntheses conducted. If meta-analysis was done, present for each the summary estimate and its precision (e.g. confidence/credible interval) and measures of statistical heterogeneity. If comparing groups, describe the direction of the effect. | Results section (Results of syntheses subsection: narrative only; no meta-analysis; qualitative direction of effects described). |
|  | 20c | Present results of all investigations of possible causes of heterogeneity among study results. | Results section (Results of syntheses subsection: heterogeneity discussed narratively by species/model) |
|  | 20d | Present results of all sensitivity analyses conducted to assess the robustness of the synthesized results. | Results section (Results of syntheses subsection: no formal sensitivity; robustness via RoB narrative). |
| Reporting biases | 21 | Present assessments of risk of bias due to missing results (arising from reporting biases) for each synthesis assessed. | Results section (Reporting biases subsection: no formal assessment; mitigated by comprehensive search).Chapter 3 |
| Certainty of evidence | 22 | Present assessments of certainty (or confidence) in the body of evidence for each outcome assessed. | Results section (Certainty of evidence subsection: certainty discussed narratively per organ based on evidence strength). |
| **DISCUSSION** | | |  |
| Discussion | 23a | Provide a general interpretation of the results in the context of other evidence. | Discussion section (general interpretation in context of prior reviews and evidence). |
|  | 23b | Discuss any limitations of the evidence included in the review. | Discussion section (limitations of included evidence subsection) |
|  | 23c | Discuss any limitations of the review processes used. | Discussion section (limitations of review processes subsection). |
|  | 23d | Discuss implications of the results for practice, policy, and future research. | Discussion section (implications for practice/policy/research subsection). |
| **OTHER INFORMATION** | | |  |
| Registration and protocol | 24a | Provide registration information for the review, including register name and registration number, or state that the review was not registered. | NA |
|  | 24b | Indicate where the review protocol can be accessed, or state that a protocol was not prepared. | NA |
|  | 24c | Describe and explain any amendments to information provided at registration or in the protocol. | no amendments mentioned). |
| Support | 25 | Describe sources of financial or non-financial support for the review, and the role of the funders or sponsors in the review. | no funding mentioned; self-supported) |
| Competing interests | 26 | Declare any competing interests of review authors. | Competing interest’s subsection: none declared) |
| Availability of data, code and other materials | 27 | Report which of the following are publicly available and where they can be found: template data collection forms; data extracted from included studies; data used for all analyses; analytic code; any other materials used in the review. | Availability subsection: data extraction forms, extracted data, and tables available in supplementary files S2/S3) |

*From:*  Page MJ, McKenzie JE, Bossuyt PM, Boutron I, Hoffmann TC, Mulrow CD, et al. The PRISMA 2020 statement: an updated guideline for reporting systematic reviews. BMJ 2021;372:n71. doi: 10.1136/bmj.n71. This work is licensed under CC BY 4.0. To view a copy of this license, visit <https://creativecommons.org/licenses/by/4.0/>
