# Supplementary material for: Physiological significance of autocrine orexinergic signaling in extra‐hypothalamic tissues
Source: Physiol Rep. 2026 Apr 24;14(8):e70892. doi: 10.14814/phy2.70892 (PMC13109654; doi:10.14814/phy2.70892)
Supplement: Supplementary file 2 — File S2: Full Search Strategies for Each Database. [file PHY2-14-e70892-s004.docx]

**Supplementary File S2: Full Search Strategies for Each Database**

This supplementary file provides the detailed search strategies used for each electronic database in the systematic review. No date limits were applied. Searches were conducted on 20–25 November 2025 (updated 30 December 2025). The strategies combined controlled vocabulary (e.g., MeSH terms where applicable) and free-text terms for orexins/hypocretins, peripheral/extra-hypothalamic locations, and local/autocrine signaling indicators. For Google Scholar, the first 300 results were screened.

**PubMed/MEDLINE Search Strategy**

("orexin*" OR "hypocretin*" OR "OX1R" OR "OX2R" OR "HCRTR1" OR "HCRTR2") AND ("peripheral" OR "extra-hypothalamic" OR "adrenal" OR "testis" OR "prostate" OR "kidney" OR "heart" OR "myocard*" OR "cardiomyocyte*" OR "pancreas" OR "adipocyte*" OR "adipose" OR "gastrointestinal" OR "intestine" OR "skin" OR "Merkel cell" OR "muscle" OR "skeletal muscle" OR "placenta" OR "ovary") AND ("autocrine" OR "paracrine" OR "local synthesis" OR "co-expression" OR "co-localization" OR "peripheral expression")

**Web of Science Core Collection Search Strategy**

(TS=(("orexin*" OR "hypocretin*" OR "OX1R" OR "OX2R" OR "HCRTR1" OR "HCRTR2") AND ("peripheral" OR "extra-hypothalamic" OR "adrenal" OR "testis" OR "prostate" OR "kidney" OR "heart" OR "myocard*" OR "cardiomyocyte*" OR "pancreas" OR "adipocyte*" OR "adipose" OR "gastrointestinal" OR "intestine" OR "skin" OR "Merkel cell" OR "muscle" OR "skeletal muscle" OR "placenta" OR "ovary") AND ("autocrine" OR "paracrine" OR "local synthesis" OR "co-expression" OR "co-localization" OR "peripheral expression")))

**Cochrane Central Register of Controlled Trials Search Strategy**

("orexin*" OR "hypocretin*" OR "OX1R" OR "OX2R" OR "HCRTR1" OR "HCRTR2") AND ("peripheral" OR "extra-hypothalamic" OR "adrenal" OR "testis" OR "prostate" OR "kidney" OR "heart" OR "myocard*" OR "cardiomyocyte*" OR "pancreas" OR "adipocyte*" OR "adipose" OR "gastrointestinal" OR "intestine" OR "skin" OR "Merkel cell" OR "muscle" OR "skeletal muscle" OR "placenta" OR "ovary") AND ("autocrine" OR "paracrine" OR "local synthesis" OR "co-expression" OR "co-localization" OR "peripheral expression")

**Google Scholar Search Strategy**

("orexin*" OR "hypocretin*" OR "OX1R" OR "OX2R" OR "HCRTR1" OR "HCRTR2") ("peripheral" OR "extra-hypothalamic" OR "adrenal" OR "testis" OR "prostate" OR "kidney" OR "heart" OR "myocard*" OR "cardiomyocyte*" OR "pancreas" OR "adipocyte*" OR "adipose" OR "gastrointestinal" OR "intestine" OR "skin" OR "Merkel cell" OR "muscle" OR "skeletal muscle" OR "placenta" OR "ovary") ("autocrine" OR "paracrine" OR "local synthesis" OR "co-expression" OR "co-localization" OR "peripheral expression".
